# *Pseudomonas aeruginosa* Biofilm Removal from Two Kinds of Granite Commonly Found in Catering Kitchen

**DOI:** 10.1155/2020/4313908

**Published:** 2020-06-17

**Authors:** Khaddouj Amzil, Fatima Hamadi, Hassan Latrache, Rachida Mimouni, Hicham Abou Oualid, Khadija Azelmad, Aissa Saidi, Abdallah Elboulani, Mustapha Mabrouki

**Affiliations:** ^1^Laboratory of Microbial Biotechnology and Vegetal Protection, Faculty of Sciences Ibn Zohr University, Agadir, Morocco; ^2^Laboratory of Bioprocess and Bio-interfaces, Faculty of Sciences and Techniques, Sultan Moulay Slimane University, Beni Mellal, Morocco; ^3^Laboratory of Biotechnology, Materials and Environment, Faculty of Sciences, Ibn Zohr University, Agadir, Morocco; ^4^Laboratory of Industrial Engineering, Faculty of Sciences and Techniques, Sultan Moulay Slimane University, Beni Mellal, Morocco

## Abstract

The biofilm formation on the surfaces which are in direct contact with food products might lead to their contamination and, consequently, present serious health problems for the consumers. The goals of the present work were to study *P. aeruginosa* biofilm formation on two granites and to investigate the efficiency of sodium hypochlorite (NaCLO) against the same biofilm formed on these substrata using the plate count method (PCM) and epifluorescence microscopy (EP). More biofilm cells adhered to Rosa Porrino than Gris Pinhel, and the PCM method indicated that NaCLO was efficient against the biofilm installed on the Gris Pinhel at the concentration of 1.5% after 15 min of treatment, while it was not efficient against the one installed on the Rosa Porrino. By contrast, the EP showed that the biofilm persists on two granites after NaCLO treatment, at different concentrations and contact times. In addition, the surface properties of granites such as mineral composition, roughness, and physicochemical properties were determined by X-ray diffraction (XRD), scanning electron microscopy coupled with electron diffraction spectroscopy coupled with energy-dispersive X-ray spectroscopy (SEM-EDS), Fourier transform infrared (FTIR), atomic force microscopy (AFM), and contact angle measurement (CAM), respectively. The results revealed that Gris Pinhel is hydrophilic with a high roughness value and Rosa Porrino is hydrophobic with low roughness, while both of them contain the quartz, feldspar, and mica as the main dominant compositions.

## 1. Introduction

*Pseudomonas aeruginosa* is a Gram-negative, aerobic, and nonfermentative bacterium, which normally lives in a moist environment. It is an important opportunistic human pathogen capable of causing severe invasive infections to patients with cystic fibrosis, neutropenia, iatrogenic immunosuppression, or disrupted anatomical barriers [1]*. P. aeruginosa* has a high adhesion ability to different surfaces such as polystyrene [2], stainless steel, and polycarbonate [3], which consequently lead to biofilm formation. This latter is more resistant to antimicrobial agents when compared to the bacterial suspension [4]. The biofilm is defined as an assemblage of microbial cells that is firmly associated with a surface and enclosed in a matrix of Extracellular Polymeric Substance (EPS) [5]. This matrix of EPS may present from 50% to 90% of the total organic carbon of biofilms [6], and it plays an important role in the protection of biofilm cells against disinfectants by preventing its penetration inside biofilms [7]. The adhesion of the bacteria to solid surfaces is a key step for biofilm formation, and it is governed by the physicochemical properties (hydrophobicity, surfaces charge, and electron donor-acceptor) of bacteria and substratum surfaces. In addition, other factors can be involved in bacterial adhesion such as surface roughness and topography.

Domestic and industrial kitchens are the most important focuses of attention for food contamination. In such environments, cross-contamination is the responsible factor of the spread of food-associated diseases [8]. Furthermore, contaminated food contact surfaces are considered one of the outbreak causes [9]. Many studies in literature showed the ability of some bacteria to attach to the granite, such as *Pseudomonas fluorescens* [10]*, Salmonella typhimurium* [8]*, Listeria monocytogenes* [11]*, Salmonella enteritidis, Staphylococcus aureus* [12, 13], and *Staphylococcus xylosus* [13]. None of these studies have specified the type of granite studied. Based on our knowledge, there is no study about the adhesion of *P. aeruginosa* to the granite surface.

Several chemical agents have been tested against biofilm cells such as peracetic acid [12], nonoxidizing aldehyde-based biocide [14], quaternary ammonium [12], and sodium hypochlorite [12]. This last disinfectant was reported to be a potential biofilm antimicrobial agent against *Staphylococcus aureus* [15]*, Prevotella intermedia, Peptostreptococcus micros, Streptococcus intermedius, Fusobacterium nucleatum, and Enterococcus faecalis* when compared to other disinfectants [16]. The bactericidal activity of sodium hypochlorite is mainly related to the release of hypochlorous acid (HCLO). This antimicrobial agent penetrates through membranes of bacterial cells and acts by a rapid mechanism of general oxidation leading to the denaturation of the protein [17]. As a result of this, the metabolic reaction stops and leads to cell death [18]. Based on our knowledge, except for the study realized by Silva et al. [12], there are no other studies about the efficiency of disinfectants against biofilm cells formed on the granite.

The aims of the present work are to study the ability of *P. aeruginosa* to form a biofilm on two kinds of granite, commonly found in a Moroccan catering kitchen, and to investigate the efficiency of sodium hypochlorite against this biofilm under different contact times (5, 10, 15, and 30 min) and concentrations (0.5, 1, 1.5, and 2%). This disinfectant was found to be the most disinfectant used by catering Moroccan kitchen (not published investigation). In addition, the physicochemical characteristics of *P. aeruginosa* and the properties of two granites (Rosa Porrino and Gris Pinhel) were determined.

## 2. Materials and Methods

### 2.1. Isolation and Identification of Bacterial Strain

The bacterium strain used in the present study was *Pseudomonas aeruginosa*, isolated from catering services in health establishment and more specifically from a stainless steel surface, after cleaning and disinfecting procedures, and identified as described hereinafter.

Genomic DNA was extracted from pure cultures using the protocol for bacteria of the Tissue NucleoSpin® kit (MACHEREY-NAGEL, Germany). For molecular detection, the primers S-D-Bact-0341-b-S-17, 5'CCTACGGGNGGCWGCAG-3′ and S-D-Bact-0785-a-A-21, 5′-GACTACHVGGGTATCTAATCC-3′), which amplify a 464 bp fragment of the 16S rRNA gene, were used with the described protocol [19]. The PCR products were excised from the gel, purified with the Gel &PCR Clean-up of Nucleo Spin® kit, and submitted to the sequencing provider service (Bio *Basic* Inc., ON, Canada) for Sanger sequencing using the same primers as for PCR reaction.

The sequences obtained were analyzed by BLAST (Basic Local Alignment Search Tool) [20] for calculating sequence similarities. Phylogenetic analyses were done by MEGA7 [21] for homology study. The bioinformatics analysis of the sequences made it possible to identify the P11 sequence as *Pseudomonas aeruginosa*.

### 2.2. Growth Condition of Bacterial Suspension

The bacterium strain was grown at 37°C for 24 h on Luria Bertani agar (three independent cultures were prepared). The medium was made using the following components: 10 g tryptone, 5 g yeast extract, 10 g NaCl, 15 g agar, and one liter of distilled water. After 24 h of incubation, bacterial cells were scraped off the agar plates and harvested by centrifugation at 8400 ×*g* for 15 min. Cell pellets were resuspended in KNO_3_ (0.1 M) and adjusted by spectrophotometer to optical density approximately between 0.7 and 0.8 corresponding to 10^8^ CFU/ml.

### 2.3. Surfaces Preparation and Disinfection

The surfaces were cut into small slides; the size of granite Rosa Porrino and Gris Pinhel was 2 cm per 2 cm, and the thickness of the two granites was 5 mm±1 mm. These surfaces were immersed in the solution of absolute ethanol for 15 min, then rinsed three times with distilled water, and autoclaved at 121°C for 20 min [22].

The selected disinfectant for this study was sodium hypochlorite (NaCLO), at four different concentrations which are 0.5, 1, 1.5, and 2%.

### 2.4. Biofilm Formation Test

The sterilized surfaces were immersed in the Petri dishes containing the bacterial suspension, for three hours at 25°C. After that, the surfaces were rinsed three times with sterile distilled water to remove nonattached bacteria. Then, the surfaces were placed in Luria Bertani broth and incubated at 25°C for 24 h. Three replicates were carried out for each experiment.

### 2.5. Effectiveness of Sodium Hypochlorite against Biofilm Cells

After the biofilm development, the granite surfaces were rinsed three times with sterile distilled water to remove the nonadherent cells. Then, they were placed in Petri dishes containing sodium hypochlorite already prepared at diverse concentrations (0.5, 1, 1.5, and 2 %), for a contact time of 5, 10, 15, and 30 min. The surfaces were rinsed three times with sterile distilled water. They were placed after in the glass tubes containing 10 ml of sterile physiological saline and sonicated at 35 kHz for 10 min and vortexed [13, 23]. To quantify viable cells, bacteria were resuspended, serially diluted 10-fold with sterilized physiological saline, and cultured on nutrient agar at the temperature of 37°C for 24 h. Three replicates were carried out for each experiment.

### 2.6. Granite Characterization

Fourier transform infrared (FTIR) spectra of samples in KBr pellets were measured on a Bruker Vector 22 spectrometer. X-ray diffraction patterns of both granites were recorded on a Bruker AXS D-8 diffractometer using Cu-K*α* radiation in Bragg-Brentano geometry (*θ*-2*θ*). Scanning electron microscopy (SEM) micrographs were obtained using an FEI Quanta 200 microscope after carbon metallization. The topography and roughness of both granites were measured using atomic force microscopy (Nanosurf flex AFM). The measurement was carried out with an easy scan 2 controller from Nanosurf. The tapping mode (Dynamic) in an ambient air environment was used for scanning and measuring. The Ra value, which is the arithmetic mean deviation of the profile, is the most commonly used descriptor of surface roughness. The Ra value was determined using the software easy scan 2 (three replicates).

### 2.7. Physicochemical Characterization of the Bacterium and Substratum Surfaces

The bacterial suspension was filtered using 0.45 *µ*m cellulose acetate filter (Sartorius) by a first washing of the filter with 10 ml of distilled water for wetting, and then 10 ml of the cell suspension was added to obtain a thick lawn of cells after filtration using negative pressure.

The free energy of bacterium and support surfaces was determined using a goniometer (GBX instruments) by the sessile drop method according to Busscher [24]. The surface energy of bacterium and substratum was determined by measuring the contact angle with three liquids: water, formamide, and diiodomethane. The hydrophobicity of the surfaces was obtained after contact angle measurement (CAM) using Van Oss et al.'s approach [25]. The hydrophobicity of the given material (i) is expressed as the free energy of interaction between two entities of that material, when immersed in water (ΔG_iwi_ (mJm^−2^)). If Δ*G*_iwi_ < 0, the material is considered hydrophobic, while if Δ*G*_iwi_ > 0, the material is hydrophilic. Δ*G*_iwi_ can be calculated through the following equation:

(1) $$\begin{array}{c} \Delta G_{\text{iwi}}=-2\left(\sqrt{\gamma_{S}^{\text{LW}}}\;-\sqrt{\gamma_{L}^{\text{LW}}}\right)+4\left(\sqrt{\gamma_{S}^{+}\times \gamma_{W}^{-}}+\sqrt{\gamma_{S}^{-}\times \gamma_{W}^{+}}\;-\;\sqrt{\gamma_{S}^{+}\times \gamma_{S}^{-}}\;-\;\sqrt{\gamma_{W}^{+}\times \gamma_{W}^{-}}\right), \end{array}$$

where *γ*^LW^ is the Lifshitz-van der Waals component, and *γ*^+^ is the electron acceptor, while *γ*^−^ is the electron donor component.

The contact angles of the three liquids are calculated using the following equation:

(2) $$\begin{array}{c} \text{Cos}\theta \;=-1\;+\frac{2\left(\gamma_{S}^{\text{LW}}\times \gamma_{L}^{\text{LW}}\right)\left(1/2\right)}{\gamma_{L}}+2\frac{\left(\gamma_{S}^{+}\times \gamma_{L}^{-}\right)}{\gamma_{L}}+2\frac{\left(\gamma_{S}^{-}\times \gamma_{L}^{+}\right)}{\gamma_{L}}, \end{array}$$

where *S* and *L* denoted solid surface and liquid phases, respectively.

### 2.8. Epifluorescence Microscopy Analysis

The surfaces colonized with *Pseudomonas aeruginosa* biofilm and the one treated with sodium hypochlorite (NaCLO) were analyzed using epifluorescence microscopy (Olympus BX41) with the following characteristics: excitation filter PB330-385 nm, dichromatic mirror of 400 nm, magnification ×100, and the scale bar 20 *μ*m. The biofilm cells were stained using 4′,6-diamidino-2-phénylindole dihydrochloride (DAPI, Germany). Each substratum was immersed in the solution of DAPI at a concentration of 71.4 *μ*M. After 10 min of incubation in the dark, the substrata were washed twice with BPS (phosphate-buffered saline) and then mounted with the immersion oil and examined with epifluorescence microscopy. The images were treated using Helicon Focus Software. The area occupied by the biofilm without and after NaCLO treatment was calculated using the software ImageJ. Five areas for each surface were included in the calculation of the average area occupied by *P. aeruginosa* biofilm without and after treatment.

### 2.9. Statistical Analyses

Statistical analyses were performed using Software STATISTICA version 6. The Newman–Keuls test was used (*P*value < 0.05). The means presented in the figures with the same letters (a/b/*c*/d) are not significantly different and the ones with distinct letters are significantly different.

## 3. Results and Discussion

### 3.1. Granite Characterization

**Figure 1:**
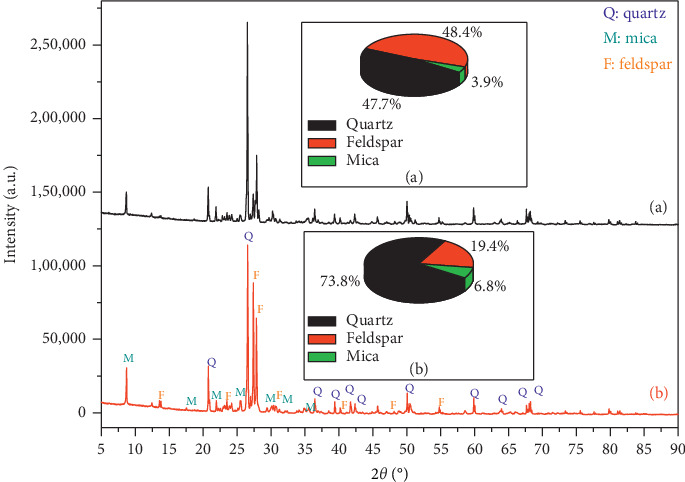
X-ray analysis of Rosa Porrino (a), Gris Pinhel (b), and mass fraction of main.

To study the elemental composition of both granite (Gris Pinhel and Rosa Porrino) samples, X-ray diffraction analysis was conducted.  Figure 1 shows the different patterns and the mass fraction histogram of principal components. As shown in the figure, quartz, feldspar, and mica were observed as the dominant compositions of both Gris Pinhel and Rosa Porrino. This result was found in [26]. The mass fraction of Gris Pinhel and Rosa Porrino was estimated and represented in the pie chart (Figures 1(a) and 1(b), respectively). As shown in the pie chart (a) and (b), the mass fractions of the samples are not similar. It could be observed that the mass fraction of quartz and feldspar in the Rosa Porrino granite is relatively similar (47.7%, 48.4%) with a small amount of mica (3.9%). However, the quartz (73.8%) is the most important in the case of Gris Pinhel, and only 19.4% and 6.8% of feldspar and mica, respectively, were found.

**Figure 2:**
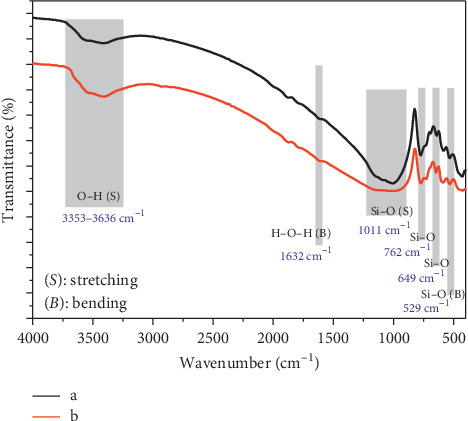
XRD analysis and FTIR analysis of ground Rosa Porrino (a) and Gris Pinhel (b).

As a part of structural characterization, FTIR analysis of granite samples was also conducted.  Figure 2 exhibits the spectra of Gris Pinhel and Rosa Porrino. It can be shown that both granite samples exhibit mainly several functional groups; indeed, the band at about 3353–3636 cm^−1^ corresponds to the hydroxyl stretching group [27]. The pic at 1632 cm^−1^ corresponds to H-O-H bending vibrations [28]. On the other hand, the pics at 1011cm^−1^correspond to Si-O stretching vibrations. The pics at 762 and 649 cm^−1^ correspond to the Si-O vibrations of quartz and 529 cm^−1^ correspond to O-Si-O bending vibrations [27]. In conclusion, FTIR analysis confirmed qualitatively the existence of Si components such as quartz, feldspar, and mica previously shown by the XRD analysis. It should be noted that the broadband corresponding to the hydroxyl group of Rosa Porrino sample is a little large compared to the Gris Pinhel sample, which confirms the existence of a large number of Si-OH groups on the surface.

**Figure 3:**
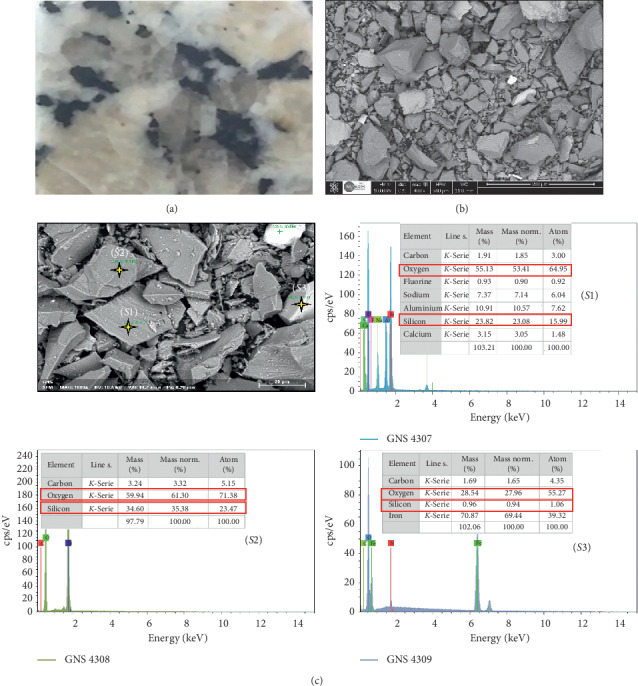
Optical image (a), SEM images (b) and (c), and corresponding local EDS analysis in different sides (S1, S2, and S3) of Gris Pinhel sample.

**Figure 4:**
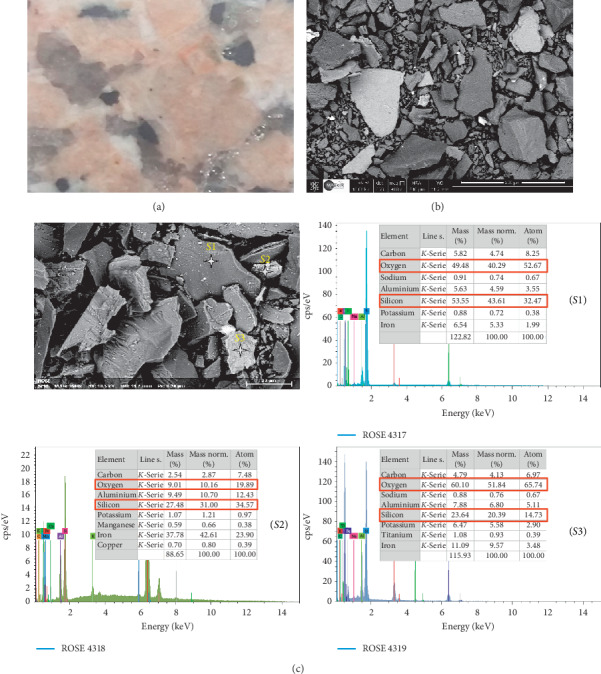
Optical image (a), SEM images (b) and (c), and corresponding local EDS analysis in different sides (S1, S2, and S3) of Rosa Porrino sample.

To examine the surface side of the granite sample as well as the elemental composition of both Gris Pinhel and Rosa Porrino granites, scanning electron microscopy and EDS analysis were carried out. The results are illustrated in Figures 3 and 4 for Gris Pinhel and Rosa Porrino, respectively. As can be observed, the optical image (Figure 3(a)) of the Gris Pinhel is gray in color, with dark spots on the surface. In the case of Rosa Porrino granite, the color is pink with black and gray spots (Figure 4(a)). The SEM images for both ground Gris Pinhel and Rosa Porrino granites show that both of them are nonspherical without any porosity. On the other hand, local EDS analysis was conducted in different spots (*S*1, *S*2, and *S*3) from Figures 3(c) and 4(c) for Gris Pinhel and Rosa Porrino, respectively. It was found that silicon and oxygen are the main elements in both samples, which is normal due to the existence of quartz and other silicon components (feldspar and mica) on the surface. Moreover, EDS analysis in different spots confirms that both Gris Pinhel and Rosa Porrino granite surfaces are heterogeneous.

### 3.2. Substratum Surface Characterization

**Figure 5:**
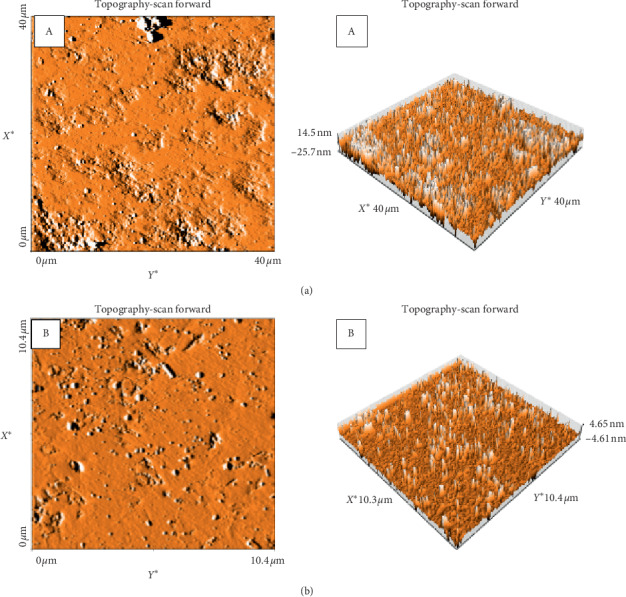
Topography (3D images) of granite Gris Pinhel (a) and granite Rosa Porrino (b).

Both granites were analyzed using atomic force microscopy.  Figure 5 presents the topography and three-dimensional images of Gris Pinhel and Rosa Porrino.  Table 1 shows the obtained results of Ra in the nanometer, which presents the mean distance of the roughness profile to the center plane of the profile. The roughness value of granite Gris Pinhel is higher than the one of Rosa Porrino. The first granite's value is in agreement with that found by Silva et al. [11] (13.1 ± 2.3 nm), which is almost the same, whereas our findings were not in agreement with the reports of Azelmad et al. [13] which showed that the roughness of the granite Noir Galaxy is 9.1 ± 3 nm. This difference could be due to the kind of substratum. In addition, Teixeira et al. [8] also determined the roughness of the granite by using AFM and their result (43.9 ± 17.9 nm) was higher than ours for both granites. The roughness was found to be one of the most important factors in surface properties that certainly affect the adhesion of bacteria [29].

### 3.3. Physicochemical Characteristics of the Bacterium and Substratum Surfaces

In the present study, the physicochemical characteristics of *P. aeruginosa* and of two kinds of granite (Rosa Porrino and Gris Pinhel) were determined by using CAM. The results are presented in Table 2. The granite Gris Pinhel has a hydrophilic character (ΔG_iwi_ = 24.45 > 0 mJ m^2^) due to the existence of a large number of Si-OH groups on the surface, while less number was remarked on the surface of Rosa Porrino which explains its hydrophobic character (ΔG_iwi_ = -63, 47 < 0 mJ m^−2^). In addition to that, the strong electron donor character of the granite Gris Pinhel could also be explained by the large number of hydroxyl groups that existed on this surface compared with Rosa Porrino with the fewer ones which explains its low electrons' donor character. Silva et al. [11] determined the physicochemical characteristics of the granite surface. According to their study, the granite surface is hydrophilic and predominantly electron donor. This result agrees with our findings concerning the physicochemical characteristics of granite Gris Pinhel and in disagreement with the ones of granite Rosa Porrino. In addition, Teixeira et al. [8] also showed that the granite surface is hydrophilic which is also in agreement with the result obtained for granite Gris Pinhel. It is important to note that both studies [8, 11] did not specify the type of granite evaluated. In addition to that, *P. aeruginosa* presents the hydrophilic surface with a higher value of electron donor and a lower electron acceptor (Table 2). This hydrophilic character could be explained by the fact that *P. aeruginosa* possesses the B-band LPS as reported by Makin and Beveridge [30]. Our results are in agreement with reports of other authors [31], which also found that *P. aeruginosa* is hydrophilic. On the other hand, Li et al. [32] and Vanhaecke et al. [33], indicated that *P. aeruginosa* has a hydrophobic character. The contradiction with our findings might be due to the difference in experimental conditions such as the temperature of stain culture which was 26°C for Li et al. [32], while it was 37°C in our study. Vanhaecke et al. [33] have determined the hydrophobicity of fifteen isolates of *P. aeruginosa* using four methods including bacterial adhesion to hydrocarbon (BATH) and CAM. Their results showed that three isolates of *P. aeruginosa* appeared hydrophilic with the BATH test and the majority of the isolates showed a hydrophobic character by using CAM.

### 3.4. Biofilm Formation of *P. aeruginosa* on Two Kinds of Granite

**Figure 6:**
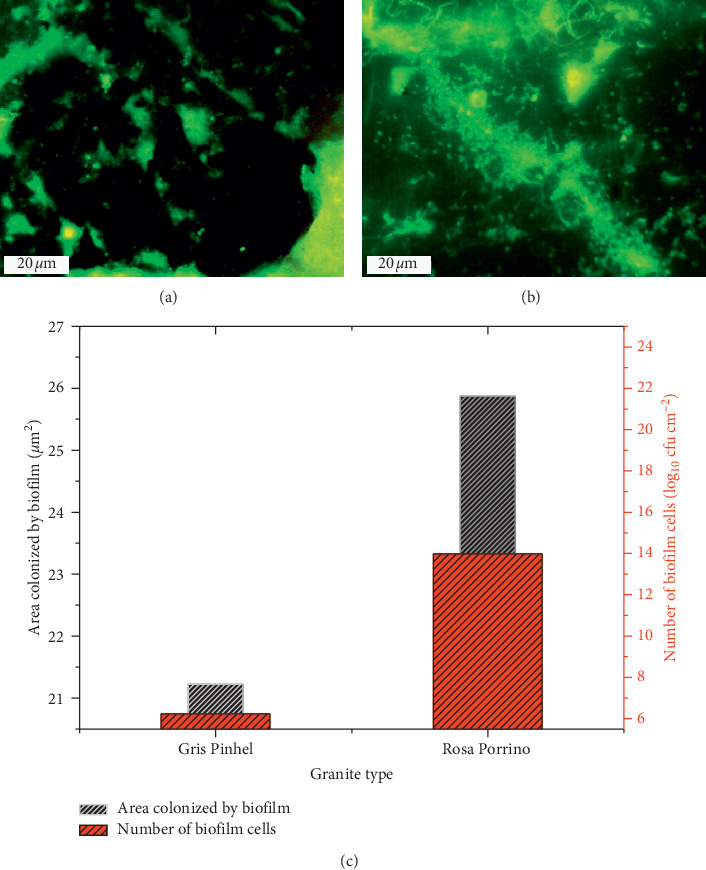
*P. aeruginosa* biofilm formation on the Gris Pinhel (a) and Rosa Porrino (b). (c) Number of biofilm cells (red bar) and the percentage of the area colonized by *P. aeruginosa* biofilm on surfaces (black bar) on granite Rosa Porrino and granite Gris Pinhel.

The ability of *P. aeruginosa* to adhere to granite Rosa Porrino and to granite Gris Pinhel with subsequent biofilm formation was investigated. The number of biofilm cells was determined through the PCM method showing that *P. aeruginosa* cells adhere more to Rosa Porrino than Gris Pinhel with a higher viable cell in the first substratum (Figure 6(c) (black bar)). The coverage area was also quantified using the EP analysis in which a significant difference (*P* < 0.05) between two granites was announced, where it was more important on the surface of the Rosa than the one observed on the Gris (Figure 6(c) (red bar)). Additionally, the surface coverage can be easily observed through the fluorescent green (Figures 6(a) and 6(b)), showing that *P. aeruginosa* biofilm colonized a large extent on the granite Rosa where just some bacterial cells are visible since they are embedded in EPS matrix that does not allow their clear visualization especially in the case of granite Gris Pinhel. This difference could be explained by the high number of *P. aeruginosa* cells that initially adhered to Rosa (5.32±0.35 Log_10_ CFU/cm^2^) after three hours of attachment step, while the low number was remarked on the Gris Pinhel (4.79±0.58 Log_10_ CFU/cm^2^). Furthermore, the physicochemical properties of the surfaces, as well as the roughness, could also have an effect on biofilm formation. *P. aeruginosa* possesses a strong electron donor and the Rosa Porrino surface has the lower electron donor and the moderate electron acceptor whereas Gris Pinhel surface has a high electron donor and a low electron acceptor. This indicates that the attraction between *P. aeruginosa* and Rosa Porrino could be more important than the one between *P. aeruginosa* and Gris Pinhel. On the other hand, the hydrophilic/hydrophobic character of the bacterium and one of two granites might not explain the existence difference in numbers of adhering bacteria to these supports; the bacterium has a hydrophilic surface and it adhered more to granite Rosa Porrino which is hydrophobic than granite Gris Pinhel which is hydrophilic. Oliveira et al. [31, 34] reported that, in an aqueous medium, adhesion is favored between hydrophobic surfaces, which can enter into closer contact by squeezing the water layer between them. Furthermore, roughness, topography, and surface chemistry can affect bacterial adhesion [13, 35] and consequently biofilm formation. Bohinc et al. [35] reported that the number of adhering bacteria increases with the increasing of the surface roughness, which is in disagreement with our results; granite Gris Pinhel has a higher roughness and a fewer number of bacteria adhering to it compared to granite Rosa Porrino with the lowest roughness. By contrast, Li et al. [32] and Flint et al. [36] reported that bacterial adhesion is not affected by surface roughness.

These data underlined that the numbers of adherent cells are affected by the kind of the surface, which is in agreement with the results obtained by DeVere et al. [37] reporting that wood and Microban incorporated plastic had a significant effect on bacterial adhesion, while glass and plastic had a little effect. In contrast, our results are in disagreement with those found by other studies which reported that surface type has no effect on bacterial adhesion [3, 38]. Abdallah et al. [3] reported that the final biomass of the 24 h biofilm was not significantly affected by the change of the surface type (stainless steel and polycarbonate).

### 3.5. Effectiveness of Sodium Hypochlorite against *P. aeruginosa* Biofilm Formed on the Granite Gris Pinhel and Granite Rosa Porrino

**Figure 7:**
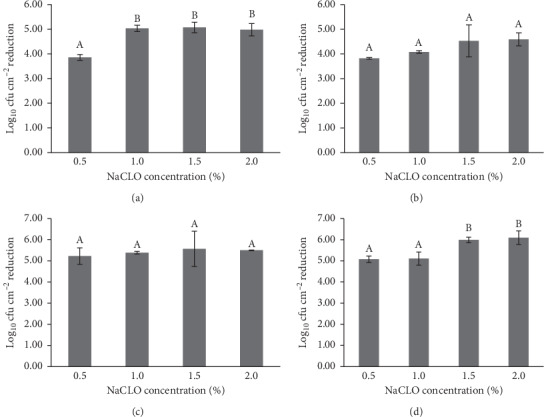
Number of biofilm cells reduced on the Rosa Porrino after 5 (a), 10 (b), 15 (c), and 30 min (d) of sodium hypochlorite treatment.

**Figure 8:**
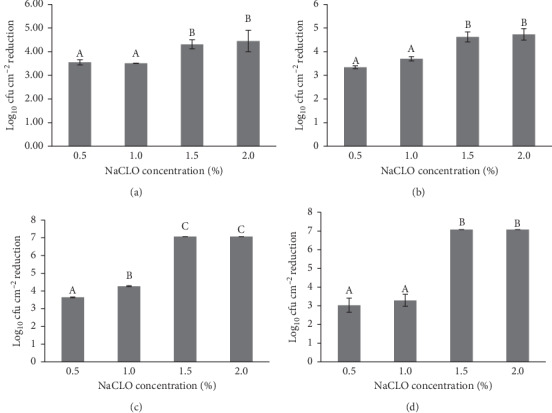
Number of *P. aeruginosa* biofilm cells reduced on the granite Gris Pinhel after 5 (a), 10 (b), 15 (c), and 30 min (d) of sodium hypochlorite treatment.

The efficiency of sodium hypochlorite against *P. aeruginosa* biofilm formed on two granites (Gris Pinhel and Rosa Porrino), under different conditions, was investigated. The results of the PCM method, which evaluated the efficiency of NaCLO against *P. aeruginosa* biofilm cells, are presented in Figures 7 and 8. In general, the number of biofilm cells reduced was increased after sodium hypochlorite treatment for both granites at different contact times (5, 10, 15, and 30 min) and concentrations (0.5, 1, 1.5, and 2%). For granite Rosa Porrino (Figure 7), there is a significant difference (*P* > 0.05) in the number of biofilm cells reduced at the concentration of 0.5 % after 5, 10, 15, and 30 min of treatment, reaching 3.86, 3.82, 5.23, and 5.08 of log_10_ reduction, respectively, while when increasing the concentrations of sodium hypochlorite to 1 %, the significance was noted only after 5 min of treatment, but when increasing it to 1.5 %, the significance was observed after 30 min. By contrast, no significance was pronounced when increasing the concentration to 2 % after 5, 10, 15, and 30 min of NaCLO treatment. For Gris Pinhel (Figure 8), there is a significant difference in the number of biofilm cells reduced at 0.5 % of NaCLO after all contact times, representing 3.55 (5 min), 3.34 (10 min), 3.64 (15 min), and 3.03 (30 min) of log_10_ reduction. Except for 15 min of contact time, no significance was pronounced when increasing the concentration to 1 %. However, the increase of NaCLO concentration to 1.5 % shows a significant difference after all contact times are tested, where the reduction has reached an important level (no colonies were observed following plate counting) after 15 min and 30 min of treatment with 1.5and 2% of NaCLO. These findings showed that sodium hypochlorite appeared to be efficient against *P. aeruginosa* that adhered to granite Gris Pinhel at the concentration of 1.5% and 2% after 15 min, but it was less efficient against the one formed on the granite Rosa Porrino whatever the time and the concentration.

**Figure 9:**
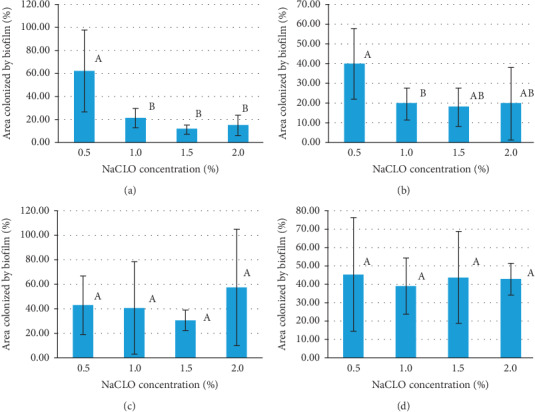
The area colonized by *P. aeruginosa* on the granite Rosa Porrino (in percentage) after 5 (a), 10 (b), 15 (c), and 30 min (d) of NaCLO treatment.

**Figure 10:**
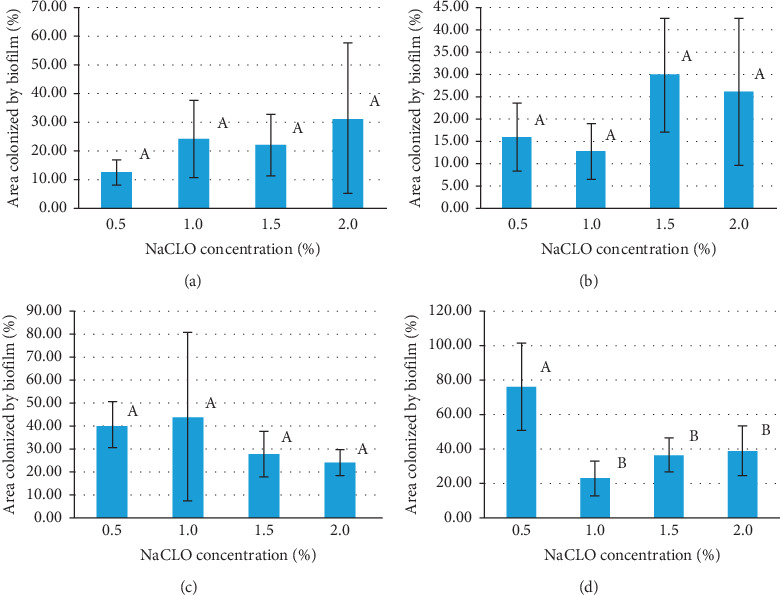
The percentage of the area colonized by *P. aeruginosa* on the granite Gris Pinhel after 5 (a), 10 (b), 15 (c), and 30 min (d) of NaCLO treatment.

**Figure 11:**
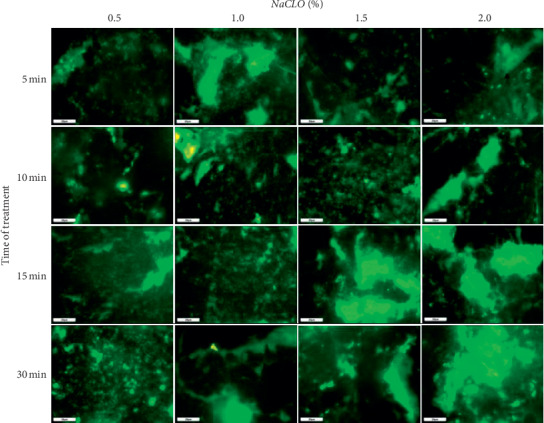
Effectiveness of NaCLO against *P. aeruginosa* biofilm installed on the granite Gris Pinhel at the concentration of 0.5, 1, 1.5, and 2% after 5, 10, 15, and 30 min of treatment.

**Figure 12:**
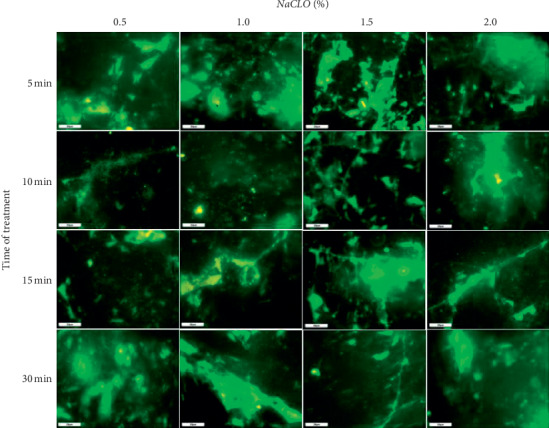
Effectiveness of NaCLO against *P. aeruginosa* biofilm installed on the granite Rosa Porrino at the concentration of 0.5, 1, 1.5, and 2% after 5, 10, 15, and 30 min of treatment.

The surface coverage after NaCLO treatment was quantified through the EP analysis (Figures 9 and 10). In the case of granite Rosa, except for the treatment after 5 min, no significant difference was pronounced after 10, 15, and 30 min when increasing the concentration up to 1, 1.5, and 2%. Similar results were obtained for Gris Pinhel after 5, 10, and 15 min, while significance was noted after 30 min of treatment when increasing the NaCLO concentration. These findings showed that *P. aeruginosa* biofilm persists on both granites after treatment at different contact times and concentrations, which can be clearly confirmed through the fluorescent green observed on granite surfaces (Figures 11 and 12). In general, the PCM and the EP analyses show that NaCLO is not efficient against *P. aeruginosa* biofilm which is still attached and viable on the surface of Rosa Porrino, while in case of Gris Pinhel even though it is still attached to the surface but its viability was lost at the concentration of 1% after 15 min of treatment where three hypotheses might be given: the bacterium is still alive but lost its capacity to develop; it is injured or died.

Martin-Espada et al. [2] reported that peracetic acid is effective against *P. aeruginosa* biofilms which are formed on polystyrene surfaces, inhibiting almost 100 % of the microbial population under the concentration of 1.61 % and a contact time of 15 min. Smith et al. [39] studied the efficacy of many biocides (benzalkonium chloride, chlorhexidine gluconate, and triclosan) and found that none of them was 100% efficient against *P. aeruginosa* biofilm. In addition, Silva et al. [12] also evaluated the effectiveness of the cleaning and disinfecting procedures in controlling *Staphylococcus aureus*, *Salmonella enteritidis*, and *Pseudomonas fluorescens* adhering to granite and stainless steel. Their results showed that chemical and mechanical actions during the cleaning procedures were more efficient against *S. aureus* adhering to stainless steel and less efficient against *P. fluorescens* and *S. Enteritidis* adhering to stainless steel and granite. They also concluded that peracetic and sodium hypochlorite were more effective than the quaternary ammonium compound against adherent cells. In addition to that, Silva et al. [12] also reported that sodium hypochlorite was more effective against adherent cells compared to other disinfectants. These findings are in agreement with those found by Abdallah et al. [3] who concluded that the resistance of *Pseudomonas aeruginosa* and *Staphylococcus aureus* biofilm to disinfectant is influenced by type surface.

In the present study, the sodium hypochlorite was more effective against *P. aeruginosa* biofilm formed on the granite Gris Pinhel which has a higher roughness value and less efficient on biofilm formed on Rosa Porrino with a lower roughness value. Frank and Chmielewski [40] reported that there is no correlation between surface roughness and *Pseudomonas* sp. biofilm removal, while Chaturongkasumrit et al. [41] indicated a correlation between Ra value and *L. monocytogenes* susceptibility to cleaning and sanitizing agents, by reporting that sanitizing with Tego-51 is efficient approximately 100 times higher on new belts when compared with the old ones, which indicates that the rough surfaces are hard to clean.

## 4. Conclusions

In conclusion, the surface properties (hydrophobicity, roughness, and electron donor character) of the granite Rosa Porrino and granite Gris Pinhel affect the attachment or adhesion of the biofilm of *P. aeruginosa* cells. Indeed, more biofilm cells developed on the granite Rosa Porrino than granite Gris Pinhel. On the other hand, sodium hypochlorite was less efficient against the viability of biofilm cells formed on the granite Rosa Porrino; however, the numbers of cells have been decreased especially after 15 min and 30 min at the concentrations of 1.5% and 2% reaching 7.07 log_10_ reduction. The same disinfectant was found to be efficient against *P. aeruginosa* biofilm installed on the granite Gris Pinhel under the same conditions. By contrast, the EP images showed that the biofilm persists on both granites after NaCLO treatment at different contact times and concentrations applied. As demonstrated by different physicochemical characterizations, the results showed that the surface properties have an effect on biofilm formation as well as on the viability of biofilm cells after NaCLO treatment which failed in removing the biofilm presented on both granites. And it is worth making efforts to develop the antiadhesive layers to stick to granite surfaces in order to eliminate or at least minimize the bacterial adhesion and consequently the biofilm formation.

## Figures and Tables

**Table 1:** Roughness values of Gris Pinhel and Rosa Porrino.

Granite sample	Roughness (nm)
Gris Pinhel	12.40 ± 2.17
Rosa Porrino	1.91 ± 0.98

**Table 2:** Contact angles, surface tension parameters (Lifshitz-van der Waals component(*γ*^LW^); electron acceptor (*γ*^+^), electron donor component (*γ*^−^)), and hydrophobicity (Δ*G*_iwi_). The means ±SD for three replicates are given.

*P. aeruginosa* and granites	Contact angles (°)			Surface tension: components and parameters (mJ m^−2^)			Hydrophobicity of cell surfaces
	*θ* diiodomethane	*θ* formamide	*θ* water	*γ* ^*LW*^	*γ* ^+^	*γ* ^−^	Δ*G*_*iwi*_ (mJ m^−2^)
Granite Rosa Porrino	71.17±0.23	102.50±1.15	117.07± 0.45	22.23±0.09	1.60±0.06	0.67±0.09	−63.47
Granite Gris Pinhel	64.40±0.23	58.30± 0.17	53.00±0.45	26.10± 0.07	0.20±0.03	40.90±0.55	24.45
*Pseudomonas aeruginosa*	70.07±0.86	47.37±0.35	25.33± 0.31	22.83± 0.50	1.00±0.10	69.1±0.85	52.90

## Data Availability

The datasets generated and/or analyzed during the current study are available from the corresponding author upon reasonable request.
